# Investigating Care Dependency and Its Relation to Outcome (ICARE): Results From a Naturalistic Study of an Intensive Day Treatment Program for Depression

**DOI:** 10.3389/fpsyt.2021.644972

**Published:** 2021-10-18

**Authors:** Sarah Glanert, Svenja Sürig, Ulrike Grave, Eva Fassbinder, Sebastian Schwab, Stefan Borgwardt, Jan Philipp Klein

**Affiliations:** ^1^Department of Psychiatry and Psychotherapy, University of Luebeck, Luebeck, Germany; ^2^Department of Psychiatry and Psychotherapy, Christian-Albrechts-University Kiel, Kiel, Germany

**Keywords:** care dependency, cognitive behavioral analysis system of psychotherapy (CBASP), metacognitive therapy (MCT), depression, adverse effects, side effects

## Abstract

**Background:** This study explores the association of experienced dependency in psychotherapy as measured with the CDQ (Care Dependency Questionnaire) and treatment outcome in depression. Furthermore, the course of care dependency and differences in the CDQ scores depending on the received type of treatment, MCT (metacognitive therapy), or CBASP (cognitive behavioral analysis system of psychotherapy), were investigated.

**Methods:** The study follows a prospective, parallel group observational design. Patients suffering from depression received an 8-week intensive day clinic program, which was either CBASP or MCT. The treatment decision was made by clinicians based on the presented symptomatology and with regard to the patients' preferences. The patients reported depressive symptoms with the QIDS-SR16 (Quick Inventory of Depressive Symptomatology) and levels of experienced care dependency with the German version of the CDQ on a weekly basis. Mixed-model analyses were run to account for the repeated-measures design.

**Results:** One hundred patients were included in the analyses. Results indicate that higher levels of care dependency might predict a less favorable outcome of depressive symptomatology. Levels of care dependency as well as depressive symptoms decreased significantly over the course of treatment. There was no significant between-group difference in care dependency between the two treatment groups.

**Conclusion:** The results suggest that care dependency might be associated with a worse treatment outcome in depressed patients. In general, care dependency seems to be a dynamic construct, as it is changing over time, while the levels of care dependency seem to be independent from the received type of treatment. Future research should continue investigating the mechanisms of care dependency in a randomized controlled design.

**Clinical Trial Registration:** https://www.drks.de/drks_web/, identifier: DRKS00023779.

## Introduction

Dependency from another is a natural phenomenon that can be found in many species, especially in humans. As we are able to develop stable relationships, we learn that “from a secure personal base […], an adult goes out to explore and […] returns from time to time,” when feeling insecure, fragile, or threatened [(1), p. 46]. Bowlby (1) further states that the deeply grounded feeling of a stable attachment figure is necessary for a confident, autonomous functioning over the whole life span, regardless of age. Thus, as described above, a certain degree of dependency seems to be indispensable in living an autonomous life that is accompanied by spontaneous actions of the individual.

On the other hand, just the word dependency itself is often associated with a negative trait, that—if it becomes too intense—may harm relationships and even cause psychiatric disorders, such as a dependent personality disorder that is characterized by a persistent, excessive craving of being supported in different areas of live, including relationships and resulting in submissiveness (2). The therapeutic relationship between patient and therapist has been described as essential for a desirable outcome (3, 4). Considering that therapist and patient spend a considerable amount of time together with the patient opening up about sensitive topics, the question arises whether dependency may evolve in psychotherapy as well and how it may affect the outcome of treatment.

This question has only rarely been addressed. Dependency has been regarded as one facet of adverse effects of psychotherapy (5), but several reviews showed that adverse events were rarely reported at all. Jonsson et al. (6) stated that only one-fifth of 132 trials reported that they monitored adverse events and even fewer actually reported adverse events. More recently, in a systematic review of 60 studies that were reported in 126 publications, it was also found that adverse events were insufficiently reported in randomized trials on persistent depressive disorder (7). These findings are in line with observations from other researchers (8–11). Additionally, the terminology of adverse events and the way how they are measured if noted at all is differing as well (6, 12, 13).

Looking at dependency as one specific aspect of adverse effects in psychotherapy, literature review is very limited. Bornstein and Bowen (14) noted earlier that there are a number of studies that assumed a correlation between dependency and depression (15), as well as other conditions such as eating disorders, anxiety disorders, alcoholism, and psychosomatic disorders (16–18). More recently, dependency was identified as a possible risk factor in psychotherapy (19, 20). Furthermore, dependency has been associated with characteristics of the patients such as passive and helpless stance (21). However, there are certain studies that gave indications for a positive effect of dependency (13, 22). Lately, Geurtzen et al. (21) addressed this problem more systematically by developing an instrument, the Care Dependency Questionnaire (CDQ) that reliably allows the measurement of the experienced dependency (23). In their first two studies utilizing the CDQ, different observations have been made. They found a positive correlation between the severity of symptoms and care dependency in a sample with 742 patients suffering from various psychiatric disorders (21), while this was not appearing in their second study with a group of students in clinical training (23). In the second study, the authors found no significant correlation between care dependency and the treatment outcome. Instead, they found a positive association between care dependency and the therapeutic alliance. A better therapeutic alliance in turn has been identified as a variable that supports a better treatment outcome (24). These findings suggest that dependency might even play a positive role for treatment success.

Besides the question *if* dependency is affecting treatment outcome, little effort has been made to understand *how* it develops over time. With regard to the development of care dependency, the aforementioned authors found that certain aspects of dependency decreased over the therapy sessions (23). On a broader view, Schneibel et al. (25) investigated the development of adverse events in group psychotherapy and found a general decrease of unwanted events and adverse treatment reactions, as measured by the questionnaire Unwanted Events and Adverse Treatment Reactions (UE-G), which examines negative implications of group therapy regarding content, size, repercussions, other patients, and the therapist (26). The authors found a general decrease of unwanted effects, which supports the idea that adverse effects may reduce over time.

Furthermore, in the recent past the question was arising, whether adverse effects in psychotherapy depend on the type of treatment. Meister et al. (27) found that patients in supportive psychotherapy reported less severe adverse events than patients who have been treated with the Cognitive Behavioral Analysis System of Psychotherapy (CBASP) by McCullough (28). In a study from (29), the authors observed that 36% of the patients treated with CBASP experienced symptom deterioration and 52% reported conflicts with the treatment team. The hypothesis arises that the intense therapeutic techniques and the intimate relationship between the therapist and the patient as a characteristic of CBASP treatment might influence the experience of negative effects during treatment. In contrast to CBASP, other existing therapeutic approaches in the treatment of depression mainly focus on reducing the typical depressive symptoms such as rumination, inhibition of drive, or loss of interests, while the specific emotional dynamics between the therapist and the patient gain less attention. One of these approaches is the metacognitive therapy (MCT) by Wells (30). To our knowledge, with regard to this type of treatment, there has been no such discussion as the one mentioned above. In sum, looking at the distinct nature of the two types of treatment, there are several differences at hand. First of all, a difference between the two treatments can be found in the nature of the individual case formulation: while in CBASP the biography of the patient builds up the basis for the following treatment, MCT is solely focusing on the current symptomatology and its related metacognitive beliefs. CBASP is further working with the intense relationship between the therapist and the patient. The therapist is disclosing his/her emotions in a disciplined way on a regular basis to help the patient experience the effect of his/her behavior. In contrast, MCT is working with a more distant relationship that is mainly focusing on the systematic reduction of depressive symptoms in a very clear and straightforward attempt while emotional situations within the sessions are not worked on in a standardized manner as in the case of CBASP. In sum, CBASP and MCT, which both represent effective treatments for depression, function in very different ways with regard to the consideration of the therapeutic relationships.

In sum, the role of care dependency remains still unclear. It is an open question whether dependency might even contribute to a successful therapy or is rather an adverse effect that impairs effective treatment. From what has been shown so far, we hypothesize that (i) a higher degree of dependency as indicated by the CDQ is associated with a less favorable outcome in depression at the end of treatment. Also, the investigation of the development of care dependency over time is of particular interest as it may offer answers to the question if CD is a construct that can be influenced and worked on in psychotherapy. Therefore, the study investigates the experienced levels of care dependency over the course of treatment (ii). Furthermore, the possibility that care dependency is dependent on the treatment type remains an unresolved issue. Thus, besides the question whether care dependency is affecting the treatment outcome, the study aims to assess whether the CDQ scores differ depending on the type of treatment received, MCT or CBASP (iii).

## Materials and Methods

The current study follows a pragmatic, prospective, parallel group observational study design. We recruited patients at the day treatment program for depression at the Department of Psychiatry and Psychotherapy, University of Lübeck, Germany, who followed an 8-week treatment of individualized and group therapy (consisting of CBASP or MCT mainly) between January 2019 and March 2020. The present study was conducted in accordance with the Declaration of Helsinki. Approval was received by the ethics committee of the University of Lübeck (ref. 17-049) and registered by German Clinical Trials Register (ref. DRKS00023779).

### Participants

**Figure 1:**
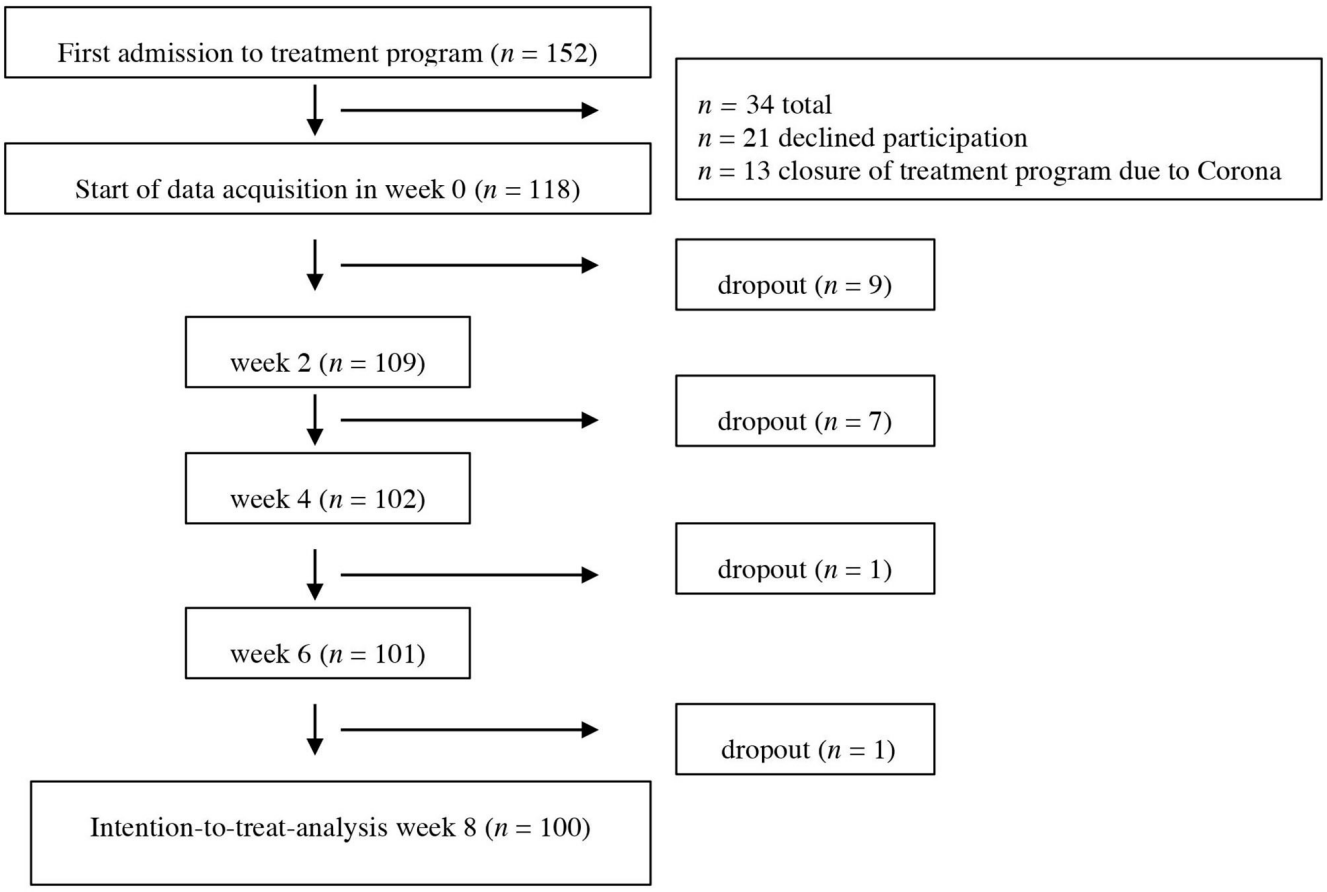
Flowchart of participants. Dropout is referring to cases that were missing data (more than 20% or missing all data points needed for analyses).

All patients admitted to the day clinic program for depression were asked to participate in the study. Almost all patients were suffering from a current depressive episode as defined by diagnostic criteria in DSM-V. Inclusion criteria were a minimum age of 18 years as well as an adequate understanding of the German language. To avoid carryover effects from previous admissions, we included only patients who have not been admitted to the treatment program within the last 12 months. Exclusion criteria for the day treatment program included acute suicidality, a history of substance use disorder, schizophrenia, delusional disorder, or bipolar disorder as well as an acute somatic illness that requires urgent treatment. Patients could only be admitted to the day clinic program if their therapist confirmed that they did not meet any of these exclusion criteria. Following the pragmatic nature of our study, we did not exclude patients from this study if they were found to meet exclusion criteria upon admission to the treatment program as long as it was clinically justifiable to treat the patient in the day clinic program. Patients did not receive any financial compensation, and all participants signed written informed consent. For an overview of the recruitment and the dropout rate, please refer to the study flowchart (Figure 1). Patients were labeled as “dropout” if they prematurely ended treatment, withdrew consent to participate in the study, or had more than 20% of missing data on the questionnaires even after repeated prompting/support to complete them.

### Intervention

All patients in the day clinic program receive intensive psychotherapy, mainly CBASP or MCT. This includes one weekly session of individual therapy by psychotherapists and three weekly sessions of group therapy by a multidisciplinary team including nurses, occupational therapists, and psychotherapists. Psychotherapists in both modalities (CBASP and MCT) were physicians in training for psychiatrist and psychotherapist, psychologists in psychotherapy training, and psychological psychotherapists. All therapists received training in both methods (CBASP and MCT) through training certified training sites. Team trainings, biweekly team supervision, and weekly supervision for therapists were mandatory. In addition, most patients received psychopharmacological treatment according to the German guidelines for depression (31); took part in occupational therapy, physical therapy, and group mindfulness exercises; and received weekly sessions with nurse specialists.

The selection of the treatment modality followed a shared-decision model and rested on three factors: diagnosis (persistent vs. episodic depression), presenting complaint (interactional problems vs. worry and rumination), and patient preference. In general, patients with persistent depressive disorder and/or primary interpersonal problems were offered CBASP while patients with major depressive disorder, anxiety disorder, or obsessive-compulsive disorder and/or primary problems of worry and rumination were offered MCT.

CBASP usually consisted of the following elements: significant other history, transference hypothesis, situational analyses during group therapy, individual therapy, and therapy administered by nurse specialists. Individual therapy included contingent personal responsivity and interpersonal discrimination exercises during individual therapy. In CBASP, therapy aims to help the patient improve his/her interpersonal skills as chronically depressed patients often have difficulties recognizing the effect their behavior has on others. Typical statements of CBASP patients in the beginning of a therapy include: “People always reject me” or “No matter what I do, I cannot change anything” (28). Improvement mainly is gained with the help of “situational analysis,” a specific tool that helps the patient to differentiate between the actual and a desired outcome in an interpersonal situation. In addition, CBASP is using techniques of disciplined personal involvement (DPI) that address occurring interpersonal situations between the therapist and the patient and include the disclosure of the therapists' positive and negative emotions triggered by the patient. These techniques help the patient to understand and experience the effect of his/her behaviors.

MCT usually consisted of case formulation, MCT group therapy, and the following techniques that were introduced in individual therapy and reinforced in therapy administered by nurse specialists: attention training technique, detached mindfulness, and worry/rumination postponement. The focus of MCT is on the development of metacognitive skills that help to prevent reoccurring worry and rumination. Typical statements of MCT patients before treatment include: “Worrying helps me to be prepared for future events.” or “I cannot control/stop the process of rumination” (30). Techniques applied include worry/rumination postponement, modifying negative and positive metacognitive beliefs, and attention training techniques.

### Assessments

During the course of treatment, patients completed various measures including the Quick Inventory of Depressive Symptomatology—Self Report (32) and the CDQ in the form of paper-pencil. The CDQ was collected in weeks 2, 4, 6, and 8. The QIDS-SR was filled out on a weekly basis from week 0 to week 8.

#### CDQ

To measure the level of care dependency, we used the revised 18-items questionnaire of Geurtzen et al. (21). The CDQ is a self-assessment questionnaire that asks the patient about his experienced degree of reliance on the therapist. In the present study, experiences as measured in the CDQ were always referring to the therapist of the individual therapy sessions even though patients had experiences with other therapists, for example in group therapies, as well. It consists of three unidimensional subscales, namely, “lack of perceived alternatives,” “submissive dependency,” and “need for contact.” All have been shown to have moderate internal consistency as indicated by Cronbach's alpha, 0.77 on average over the different time points measured. Scores of the subscales can be combined in a total scale (0.87 on average) for an encompassing assessment of perceived dependency. The total scale represents the mean of the three subscales. Items are rated on a seven-point Likert scale ranging from 1 (completely disagree) to 7 (fully agree). The instrument was translated to the German language by three experienced clinicians, following the forward–backward method (33) which is most commonly used (34). The reliability analysis based on the current dataset showed good to very good internal consistency for all subscales. Using the scores for week 2, Cronbach's alpha for “lack of perceived alternatives” was 0.76, “submissive dependency” was 0.70, and “need for contact” was 0.85. Also, for week 4, week 6, and week 8, Cronbach's alpha showed good to very good values, ranging from 0.76 to 0.82 for “lack of perceived alternatives,” 0.86 to 0.89 for “need for contact,” and 0.74 to 0.82 for “submissive dependency.”

#### Quick Inventory of Depressive Symptomatology—Self Report (QIDS-SR16)

To measure the severity of depressive symptoms over the course of treatment, we used the German version of the QIDS-SR which has shown acceptable internal consistency (Cronbach's alpha = 0.77) and a high correlation with the Beck Depressive Inventory II (BDI-II), *r* = 0.81 (35). It comprises 16 questions assessing depressive symptoms experienced during the last 7 days. Patients' score can vary between 0 and 27, with a higher score indicating a higher degree of symptom severity.

### Statistical Analyses

Statistical analyses were conducted using SPSS (IBM SPSS Statistics for Mac, version 21.0). All statistical tests were two-tailed tests with significance levels set at *p* ≤ 0.05. Pre–post effect size estimates were calculated by dividing the difference between the groups to compare by the pooled standard deviation of the two groups. Effect size measures will be interpreted as *d* = 0.2 indicating a small effect, *d* = 0.5 indicating a medium effect, and *d* = 0.8 indicating a large effect (36). Analyses were conducted using the intention-to-treat sample (ITT), which included all participants with complete baseline data irrespective of protocol deviations (e.g., meeting exclusion criteria such as current substance use disorder or history of bipolar disorder). For this analysis, individual missing values in the CDQ were replaced using the individual participant mean for the respective subscale if the number of missing items did not exceed 20% (37). Missing sum scores of the QIDS-SR and CDQ (ranging from 3 to 19%) were replaced using the mean of the posterior distribution from the fully conditional specification method obtained by iterative Markov Chain Monte Carlo estimation (38) using 10 imputations per missing value. Single cases that were missing more than 20% of data or missing complete CDQ and QIDS data were declared as dropouts and not considered in ITT (see first part of flowchart, Figure 1). Analyses to investigate differences in experienced care dependency between the two treatments included 41 patients in the CBASP group and 55 patients in the MCT group (four patients were excluded for this analysis as they received individualized Cognitive Behavioral Therapy). In order to control for the effect of repeated measures data, linear mixed models (LMM) were used. Subject ID was included as a random factor in all analyses.

#### Main Analyses: Associations of Depression and Care Dependency

For the first hypothesis (higher CDQ is associated with less favorable outcome), we ran a first model with the QIDS as dependent variable. The four different time points of the CDQ (week 2, week 4, week 6, and week 8) were used as a time-variant covariate while the received concept (CBASP vs. MCT) was used as a time-invariant covariate. Thus, the CDQ served as level 1 unit (within-subject) and the type of treatment as level 2 unit (between-subject). Additionally, we controlled for the baseline score of the QIDS.

#### Secondary Analyses: Development of Care Dependency and Its Relation to Type of Treatment

For the second (change of CDQ score during treatment) and third hypotheses (influence of the treatment concept on CDQ change), a second model was run with the CDQ scores as dependent variable. We used four different time points of the QIDS score as time-variant covariate while the therapeutic concept again was used as time-invariant covariate. In this model, the QIDS score served as level 1 unit (within-subject) and the treatment type as level 2 unit (between-subject). Here, we controlled for the baseline score of the CDQ. Each of the two models was run four times: one with the CDQ total score and one each for all of the three CDQ subscales.

#### Sensitivity Analyses

We also calculated the following sensitivity analyses. For the per protocol analysis, we included only participants who met all the inclusion and exclusion criteria. Thus, we excluded 12 participants due to a known history of substance use disorder, bipolar disorder, or delusional disorder. For a separate analysis that was aimed at increasing statistical power, we used a combined dataset which consists of the current dataset and an older dataset in the same treatment program (*n* = 75, 55% female, mean age 41.54 (SD = 14.22), ranging from 19 to 64 years. This older data set was recruited between May 2017 and March 2018 using the same in- and exclusion procedures, following the same diagnostic procedure, the same interventions, and the same assessments with two exceptions: the treatment duration was only 6 weeks, and the CDQ was collected in weeks 2 and 6 only. Accordingly, this analysis encompassed two instead of four measured observation times with regard to the CDQ (week 2 and week 6). The final dataset encompassed data of 175 individuals (48% female), mean age 41.43 (SD = 13.53), ranging from 18 to 68 years.

## Results

### Main Sample Characteristics

**Figure 2:**
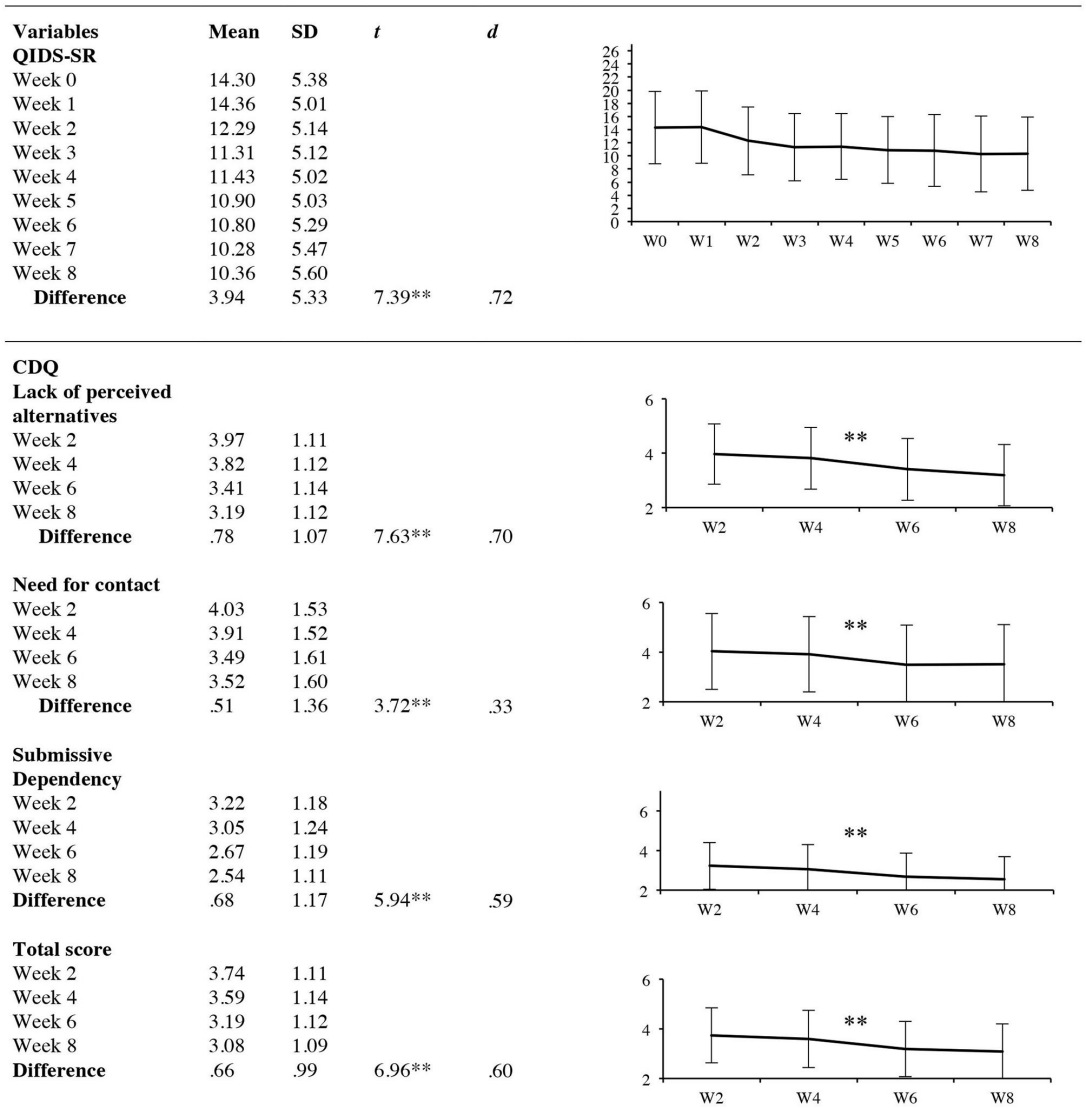
Results of secondary analyses. Development of depressive symptoms and care dependency. QIDS-SR, quick inventory of depressive symptomatology, short form; CDQ, care dependency questionnaire, effect sizes *d* were calculated for pre-post differences. *p < 0.05, **p < 0.001.

Analysis is based on the data of 100 individuals. Patients were between 18 and 68 years old (*M* = 41.3, *SD* = 13). About 43% were female, 54% were employed, and about 25% were living together in a relationship. Ninety-six percent of the patients were suffering from a current depressive episode, while half of them suffered from a persistent depressive disorder (longer than 2 years with at least some of depressive symptoms). Almost 60% described an early onset (<21 years). A total of 81% received psychotropic medication; of these, more than one-third was treated with selective serotonin reuptake inhibitors (SSRIs). For a detailed description of demographical and clinical characteristics (see Table 1). The development of depressive symptomatology during treatment is found in Figure 2. With regard to the efficacy of treatments, an ANCOVA was calculated to reveal possible differences between the two types of treatments. The results showed no significant differences in the efficacy of treatments when controlling for the QIDS baseline score, *F*_(1,94)_ = 2.78, *p* = 0.10, η_*p*_^2^ = 0.03. The symptomatic change as indicated by the QIDS change score was *M* = 4.99 (*SD* = 5.69) for CBASP and *M* = 3.07 (*SD* = 5.29) for MCT.

**Table 1:** Clinical characteristics and demographics.

	**All**		**CBASP**		**MCT**		
	***n*** **=** **100**		***n*** **=** **41**		***n*** **=** **55**		
**Clinical characteristics**	**Mean**	**SD**	**Mean**	**SD**	**Mean**	**SD**	**Test statistic U**
Age	41.34	13.05	38.12	12.91	43.39	12.97	861.00
Severity of QIDS-SR 16 week 0	14.41	5.30	15.15	4.86	13.92	5.22	1108.00
Number of depressive episodes	3.36	1.49	7.34	6.75	8.49	15.21	914.00
	***N*** **/%**		***N***	**%**	***N***	**%**	**Test statistic** **χ**^2^
Diagnoses							
PDD							
Persistent depressive episode with intermittent depressive episodes, with current episode	45		23	56.1	21	38.2	4.73
Recurrent depressive episode with current depressive episode	46		13	31.7	32	58.2	8.37^*^
First depressive episode	4		3	1.6	0	0	14.57^*^
Other	5		2	4.9	2	3.6	8.84^*^
Early onset of depression (before age of 21)	57		31	75.6	22	40	13.61^*^
Medication	81		33	81	44	80	0.98
SSRI	36		16	39	18	32.7	
Combination of AD	12		3	7.3	8	14.5	
Lithium or antipsychotic augmentation	13		4	9.8	9	16.3	
**Demographics**	***N*** **/%**		***N***	**%**	***N***	**%**	**Test statistic** **χ**^2^
Female gender	43		18	43.9	23	41.8	0.26
Marital status							10.52
Married	33		9	22	24	43.6	
Single	52		23	56.1	26	47.3	
Divorced	15		9	22	5	9.1	
Language							9.04
German	93		36	87.8	53	96.4	
Other	7		5	12.2	2	3.6	
School education							5.53
Lower	30		11	26.8	15	27.3	
Middle	32		15	36.5	18	32.7	
Higher	16		7	17.1	8	14.5	
Highest	21		8	19.5	13	23.6	
No diploma	1		0	0	1	1.8	
Employment status							7.03
Full-time	28		11	26.8	15	27.3	
Part-time	18		5	12.2	12	21.8	
Marginally	8		5	12.2	3	5.5	
Not employed	46		20	48.8	25	45.5	

*CBASP, cognitive behavioral analysis of psychotherapy; MCT, metacognitive therapy. Test statistics were computed to compare CBASP with MCT*.

* *p < 0.05*.

#### Main Analyses: Associations of Depression and Care Dependency

For the ITT, only one of the four examined subscales of the CDQ (“lack of perceived alternatives”) was significantly associated with the development of depressive symptomatology over the course of treatment, *B* = 0.44, *SE* = 0.21, *p* = 0.036. Running the same analysis for the per protocol dataset, several subscales could be identified as significant predictors for depressive symptoms: “lack of perceived alternatives” with *B* = 0.58, *SE* = 0.23, *p* = 0.010, “need for contact” with *B* = 0.38, *SE* = 0.16, *p* = 0.021, and the total score with *B* = 0.49, *SE* = 22, *p* = 0.031. Only the subscale “submissive dependency” did not reach a statistical significant level, *p* > 0.05. Running the mixed-model analysis for the combined dataset, we identified two of the three subscales as well as the total scale as potential predictors for depressive symptomatology at the end of treatment with “lack of perceived alternatives,” *B* = 0.61, *SE* = 0.19, *p* = 0.002, “need for contact,” *B* = 0.45, *SE* = 0.14, *p* = 0.001, “submissive dependency,” *B* = 0.34, *SE* = 0.17, *p* = 0.043, and the total score, *B* = 0.62, *SE* = 0.19, *p* = 0.001. For a detailed overview of all results of the different analyses for all subscales (please see Table 2).

**Table 2:** Results of multilevel-model main analysis.

**Dataset**	**Value**	**SE**	***t***	***p***
**ITT (*n*** **=** **100)**				
Lack of perceived alternatives	0.44	0.21	2.11	0.036^*^
Need for contact	0.27	0.15	1.81	0.071
Submissive dependence	0.16	0.18	0.89	0.376
Total score	0.38	0.21	1.84	0.066
**PP (*****n*** **=** **88)**				
Lack of perceived alternatives	0.57	0.22	2.58	0.010^*^
Need for contact	0.38	0.16	2.31	0.021^*^
Submissive dependence	0.14	0.19	0.76	0.446
Total score	0.48	0.22	2.17	0.031^*^
**Combined dataset (*****n*** **=** **175)**				
Lack of perceived alternatives	0.61	0.19	3.15	0.002^**^
Need for contact	0.45	0.14	3.23	0.001^**^
Submissive dependence	0.34	0.17	2.03	0.043^*^
Total score	0.62	0.29	3.25	0.001^**^

*CDQ scores as predictors for depressive symptomatology at the end of treatment. ITT, intention-to-treat analysis; PP, per protocol; SE, standard deviation*.

* *p < 0.05*,

** *p ≤ 0.001*.

#### Secondary Analyses: Development of Care Dependency and Its Relation to Type of Treatment

The LMM revealed a significant time effect for all CDQ subscales as well as the CDQ total scale, all *p* < 0.001. More precisely, *post-hoc* t-tests showed a decrease in the following subscales: lack of perceived alternatives, *t*_(99)_ = 7.63, *p* = 0.000, *d* = 0.70 (95% CI: 0.57–0.99), need for contact *t*_(99)_ = 3.72, *p* = 0.000, *d* = 0.33 (95% CI: 0.33–0.79) and submissive dependency *t*_(99)_ = 5.94, *p* = 0.000, *d* = 0.59 (95% CI: 0.44–0.91) as well as the total scale *t*_(99)_ = 6.96, *p* = 0.000, *d* = 0.60, (95% CI: 0.46–0.88). A detailed overview of the results is found in Figure 2. This effect was also found for the per protocol analysis and the combined dataset. The LMM did not show any significant differences in care dependency between the two types of treatments for the different subscales, *p* > 0.05. These results were found for all datasets (ITT, PP, combined dataset).

## Discussion

### Summary of Results

The present study gives a first insight on different aspects of the specific construct of care dependency in a group of depressed patients with regard to its possible positive or negative impact on symptom severity, the development of care dependency over the course of treatment, and whether the degree of experienced care dependency differs between two quite distinct therapeutic concepts in the treatment of depression, namely, CBASP and MCT. The results suggest that a higher degree of care dependency at the beginning of treatment might be associated with a less favorable treatment outcome. The degree of experienced care dependency decreased over the course of time while there were no differences in care dependency with regard to the two different therapeutic concepts.

In the main analysis, we found indicators of an association between care dependency and outcome when following the ITT and per protocol analysis approach. More precisely, for the ITT, the subscale “lack of perceived alternatives” appeared to be a possible predictor for the development of depressive symptoms. When excluding patients with a known history of substance use disorder, bipolar disorder, or delusional disorder in the per protocol analysis, additionally the subscales “need for contact” as well as the total scale of the CDQ reached statistical significance, possibly owing to the more homogenous sample. Due to the relatively small datasets, we combined the current dataset of this study with the dataset of an earlier iteration of the study. In this analysis, we found all the subscales to be associated with the development of depressive symptoms. Since these results only emerged on the sensitivity analyses, this needs to be confirmed in future studies.

Investigating the course of care dependency showed a clear picture for all subscales across all datasets, indicating that care dependency seems to be dynamic construct that is reducing over time. The degree of experienced care dependency seems to be independent from the received type of therapeutic treatment.

### Comparison to Existing Studies

In general, studies investigating adverse effects of psychotherapy are rare (6, 7, 9). Furthermore, as mentioned before, the incoherent picture of definitions that are used (e.g., side effects, negative effects, adverse events), and the numerous ways how they are reported, is impeding the comparison to earlier studies. As far as we know, we were the first to investigate the specific construct of care dependency over time in a clinical sample of moderately depressed patients as indicated by the Quick Inventory of Depressive Symptoms.

#### Care Dependency as a Predictor for Symptomatic Development in Depressed Patients

Taking the results of the main analysis into consideration, we found hints that aspects of care dependency might serve as potential predictors with regard to the development of depressive symptoms. These results were detected partly in the ITT and the PP and across all subscales and the total scale in the combined data analysis. For all analyses across all datasets, we found a positive direction of effects, which allows the assumption that a higher degree of experienced care dependency is associated with a higher degree of depressive symptomatology at the end of treatment. These findings are in line with results from the first study operating the CDQ by Geurtzen et al. (21) who found a higher degree of care dependency to be associated with a higher degree of symptom severity in a large cross-sectional sample of 742 outpatients with different psychiatric disorders. The negative potential of experienced dependency was also discussed and taken into consideration before (19, 20, 39, 40). However, another study from Geurtzen et al. (23) could not find the negative association with symptom development in a sample of students receiving clinical training. As the same authors mentioned, the different findings may be due to the different characteristics of the samples, patients vs. students. In sum, the question whether dependency as measured by the CDQ is beneficial for treatment outcome or not should be subject to future studies that further investigate this question in larger samples to give a better understanding of the complex construct of care dependency.

#### Development of Care Dependency

At the beginning of treatment, patients' medium answer to the CDQ items was in between “slightly disagree” (3) and “neutral” (4). This observation is close to what has been shown by the Dutch colleagues in their first CDQ study with a mixed patient sample (21), but stronger than what has been found by the same colleagues when running the study with students in clinical training for CBT (23), who scored around 2, “strongly disagree.” The differences might reflect the extent of symptom severity as well as the increased despair and the need for psychological treatment in the clinical samples. With regard to the development over time, we found a continuous decrease of care dependency over the treatment from week 2 to week 8 in all the subscales as well as the total scale. Again, studies to compare the development of adverse effects or even care dependency are scarcely available. However, the results support the view that care dependency differs from a personality trait, which is assumed to be a rather stable construct (2). Precisely, care dependency could be “elicited or reinforced by creating a specific therapeutic context” [(23), p. 10]. The researchers found no relation between dependency as a trait and care dependency.

What remains unclear is the question what actually influences the reduction of experienced feelings of care dependency. Thinking about possible factors that may influence feelings of dependency, the construct of self-efficacy inevitable comes up. Self-efficacy is defined as “people's beliefs about their capabilities to produce designated levels of performance that exercise influence over events that affect their lives” [(41), p. 71]. It can be assumed that a stronger belief of self-efficacy could reduce the feelings of dependency. The important role of self-efficacy for treatment outcome in depression has already been discussed in the late 90s (42). The authors assumed the self-efficacy theory of depression to be an additional model next to the prominent hopelessness model and Beck's cognitive model at these times in the explanation and understanding of depression. Various studies were able to show the influence of self-efficacy for a variety of somatic and psychological diseases, such as substance use disorders (43, 44), chronic low back pain (45), human immunodeficiency virus (46), posttraumatic stress disorder (47), and depression (47–51). In these studies, researchers found that self-efficacy is strongly associated or influencing the development of depressive symptoms.

When it comes to the distinct relationship between dependency and self-efficacy, the number of available studies is limited. However, Iancu et al. (52) investigated a small sample of patients suffering from social anxiety disorder and found that the social anxiety sore correlated negatively with self-efficacy and positively with dependency. This study indicates lower rates of self-efficacy and higher rates of dependency to be associated with a higher symptom severity. These results support the view that there might be a relationship between self-efficacy and dependency as well. Certainly, future studies are needed to investigate the relationship between care dependency and self-efficacy. However, it becomes clear that *if* care dependency is affecting the treatment outcome in a negative way, it should be examined which factors might influence feelings of dependency so that these can be worked on or in case of self-efficacy reinforced.

However, besides changes in self-efficacy as an internal variable that might influence feelings of dependency, one should further take external factors into consideration, too. For example, it is without doubt that psychopharmacological treatment can induce emotional and behavioral effects in patients (53, 54). These effects can be various and include feeling emotionally numb and caring less about others (55). In the recent study, about four-fifths (81%) were treated with antidepressant medication. Due to the small number of those without medication, we did not compare the two groups. However, future studies should investigate whether medication might exert an influence on care dependency. Additionally, other external variables should be taken into consideration in future studies. For example, the patients in this study received individual as well as group therapy. Even though the patient is completing the CDQ with regard to the main therapist, the question of influences of interactions with accompanying group therapists arises. So far, it is unclear if these affected the levels of care dependency toward the main therapist. It would be desirable to study care dependency in outpatient settings in particular as the possible influence of other confounding factors might be reduced and thus the specific aspects of care dependency may become more visible.

#### Secondary Analyses: Care Dependency and Its Relation to Type of Treatment

To our knowledge, there are no other studies that investigated whether care dependency differs with regard to the received treatment. Our results show that there is no difference between the two treatment groups. This could be due to a lack of statistical power and should be reinvestigated in a larger sample. Results from Klein et al. (56) indicate that CBASP is associated with a stronger therapeutic alliance compared to supportive psychotherapy. Adding the results from the Dutch colleagues (21, 23), who found a stronger therapeutic alliance to be associated with higher levels of dependency, a higher degree of experienced dependency might be reflected in CBASP compared to Supportive Psychotherapy. However, we have no information on MCT in this regard and the comparability is very limited at this point. Also, the fact that patients shared a notable amount of therapies besides the individual therapy and that these experiences possibly exerted an influence on the therapeutic experience, might have reduced differences between CBASP and MCT. Future studies that investigate outpatient settings, which are less sensitive to confounding variables such as other shared therapies or relationship building with other patients, could shed more light on this matter. Additionally, when interpreting our results with regard to the chosen treatment type, the question arises whether there might have been differences between the patients that we did not take into consideration, such as distinct personality traits. Future studies should investigate possible differences between treatment groups before and control for these in their statistical analyses. Furthermore, there appeared some significant differences with regard to the characteristics of the depressive symptomatology. More precisely, patients with an early beginning of depression were found more often in the CBASP group than in the MCT arm. It is questionable whether this difference plays a crucial role with regard to the development of care dependency. However, the fact that care dependency levels seem to be unaffected from the type of treatment could also suggest the idea that care dependency is a construct that is independent from the therapists' behavior. This should be investigated in future studies.

### Strengths and Limitations

Regarding the strengths of the study, to our knowledge, we were the first to investigate care dependency in a longitudinal study design in a clinical setting over four points in time, to explore its relation to depressive symptoms, and to explore differences in experienced care dependency in two main treatments for depression, CBASP and MCT. According to Leichsenring (57), important aspects in order to increase the ecological validity are an observational design, a dropout analysis, and pretreatment assessment. These criteria have been met. Furthermore, the study represents the reality of psychotherapeutic treatments. Another strength that should be mentioned is the high representativity of sociodemographic data in this naturalistic study, as we find an almost equal division of male and female participants, a wide range of school education, and a high number of employed people in full or part time (46%) which results in a good comparability with general population data. These characteristics support the generalizability of the findings in this study to real-world clinical settings and are comparable to general population data (58).

Still there are some limitations that require attention when interpreting our results. First of all, sample size calculation was based on estimates and issues with regard to the naturalistic design of the study, including the given fact that possible admissions to the study depend on external factors such as the available treatment capacities. However, the study is lacking an adequate sample size calculation. For our main analysis, we were imputing up to 19% of missing data. Even though we followed the recommendation of Downey and King (37), this is a strong interference in our dataset that may lead to a loss of statistical power. This interference might have been reflected in the different outcomes of our analyses. We tried to reduce this possible bias with the help of sensitivity analyses. In sum, generalizability of our findings is limited at this point in time and needs further investigation.

When investigating the development of care dependency, we did not take any other variables, such as self-efficacy, into consideration. This would have been helpful to understand the mechanism of the development and course of care dependency better. With respect to the comparison between MCT and CBASP, it has to be mentioned that the presented groups were rather small, which is a limiting factor when comparing groups. Additionally, patients were assigned to one group or the other depending on their diagnosis, the presented complains and patients' preferences. This may enhance the risk of confounding variables affecting our results (59). Even though we could not identify any problematic consequences of the chosen group allocation, future studies should focus on randomized controlled trials to reduce the potential of confounding effects. Allocating patients in a randomized way also could help to control for unobserved differences, e.g., differences with regard to the personality of the patients. In the current study, we did not control for personality variables that in turn might affect care dependency as well.

In sum, we investigated the complex construct of care dependency. We consciously took possible different aspects of care dependency into consideration as the systematic investigation of this specific construct is relatively new and the available literature appears to be very limited. Our data suggest that care dependency might play a crucial role as a predictor for symptomatic change, declines over the course of treatment but does not seem to be affected by two distinct therapeutic strategies, CBASP vs. MCT. Future research should focus on investigating care dependency as a possible predictor in randomized controlled studies.

## Conclusion

The results suggest that care dependency might negatively affect outcome in patients with depression. In general, care dependency seems to be a dynamic construct, as it is changing over time, while the levels of care dependency seem to be independent from the received type of treatment. Future research should continue investigating the mechanisms of care dependency in a randomized design to understand its potential benefits and harms for treatment outcome, to identify possible variables that influence the degree of reported care dependency and finally, to increase generalizability of the results.

## Data Availability Statement

The raw data supporting the conclusions of this article will be made available by the authors, without undue reservation.

## Ethics Statement

The studies involving human participants were reviewed and approved by University of Luebeck. The patients/participants provided their written informed consent to participate in this study.

## Author Contributions

Conception and design: JK with support from SG. Analysis of data: SG with support from SS and JK. Interpretation of data: SG and JK with support from EF. Drafting of manuscript: SG. All authors made substantial contribution to the manuscript and gave approval to the final version before submission.

## Funding

We acknowledge financial support by Land Schleswig-Holstein within the funding program Open Access Publikationsfonds.

## Conflict of Interest

JK received funding for clinical trials (German Federal Ministry of Health, Servier), payments for lectures on Internet interventions (Servier), and payment for workshops and books (Beltz, Elsevier, Hogrefe, Springer) on psychotherapy of chronic depression and psychiatric emergencies. EF received funding for clinical trials (Else Kröner-Fresenius Stiftung, University of Lübeck, Addisca gGmbH) and payments for workshops and presentations as well as for books and DVDs on psychotherapy and depression (Beltz). The remaining authors declare that the research was conducted in the absence of any commercial or financial relationships that could be construed as a potential conflict of interest.

## Publisher's Note

All claims expressed in this article are solely those of the authors and do not necessarily represent those of their affiliated organizations, or those of the publisher, the editors and the reviewers. Any product that may be evaluated in this article, or claim that may be made by its manufacturer, is not guaranteed or endorsed by the publisher.
